# Supplementary material for: The functional organization of chromosome territories in single nuclei during zygotic genome activation
Source: Sci Rep. 2026 Jan 18;16:5668. doi: 10.1038/s41598-026-35953-0 (PMC12891561; doi:10.1038/s41598-026-35953-0)
Supplement: Supplementary file 1 — Supplementary Material 1 [file 41598_2026_35953_MOESM1_ESM.pdf]

Supplementary Information

The functional organization of chromosome territories in single nuclei during zygotic genome activation

Akshada Shankar Ganesh, Taylor M. Orban, Romir Raj, Peter I. Fatzinger, Anna Johnson, Sean M. Riccard, Akhmed Zhanaidarov, Mayu Inaba, Jelena Erceg

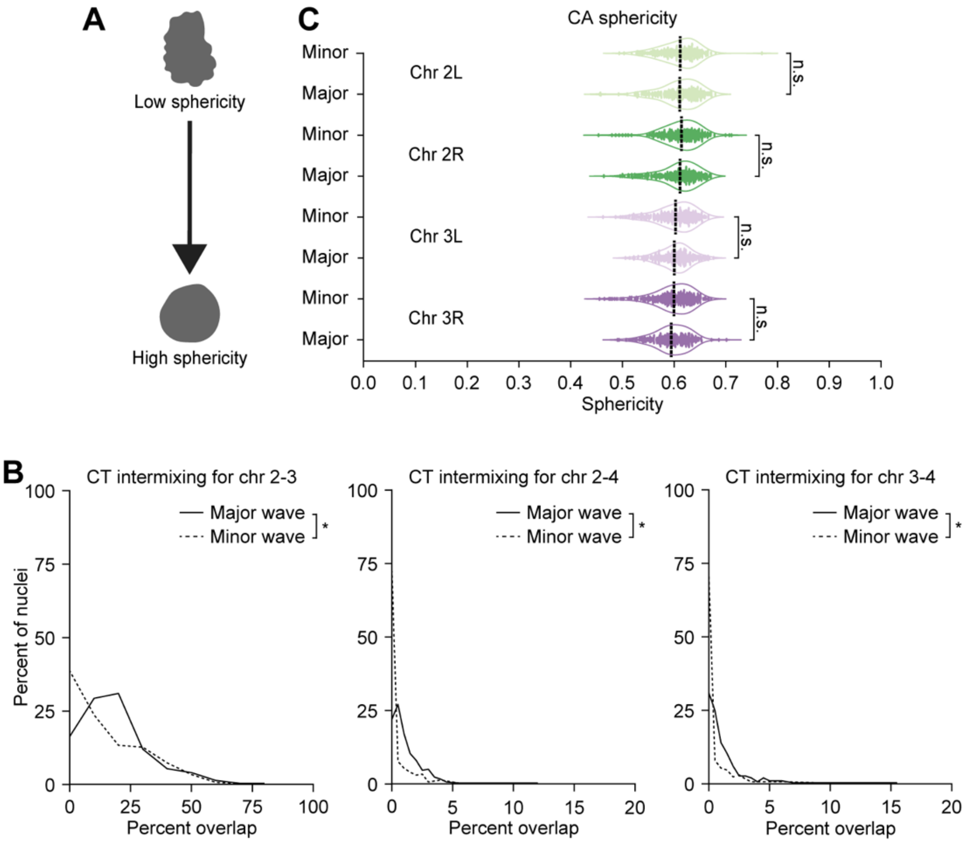

Supplementary Fig. S1. CT intermixing and sphericity of CAs.

(**A**) Illustration showing low and high sphericity. (**B**) Histogram with binned data indicating CT intermixing between chromosomes 2, 3, and 4 during ZGA. At least three replicates; n = 300 nuclei; *$p \leq 8.76\times10^{-8}$, Mann-Whitney two-sided *U* test. 2-3 and 2-4 CT overlaps as a percent of chromosome 2 CT volume; chromosome 3-4 overlap as a percent of chromosome 3 CT volume. (**C**) CA sphericity between the minor and major waves of ZGA. Chr, chromosome; median, dashed line; at least three replicates; n ≥ 300 nuclei; n.s., not significant, Mann-Whitney two-sided *U* test.

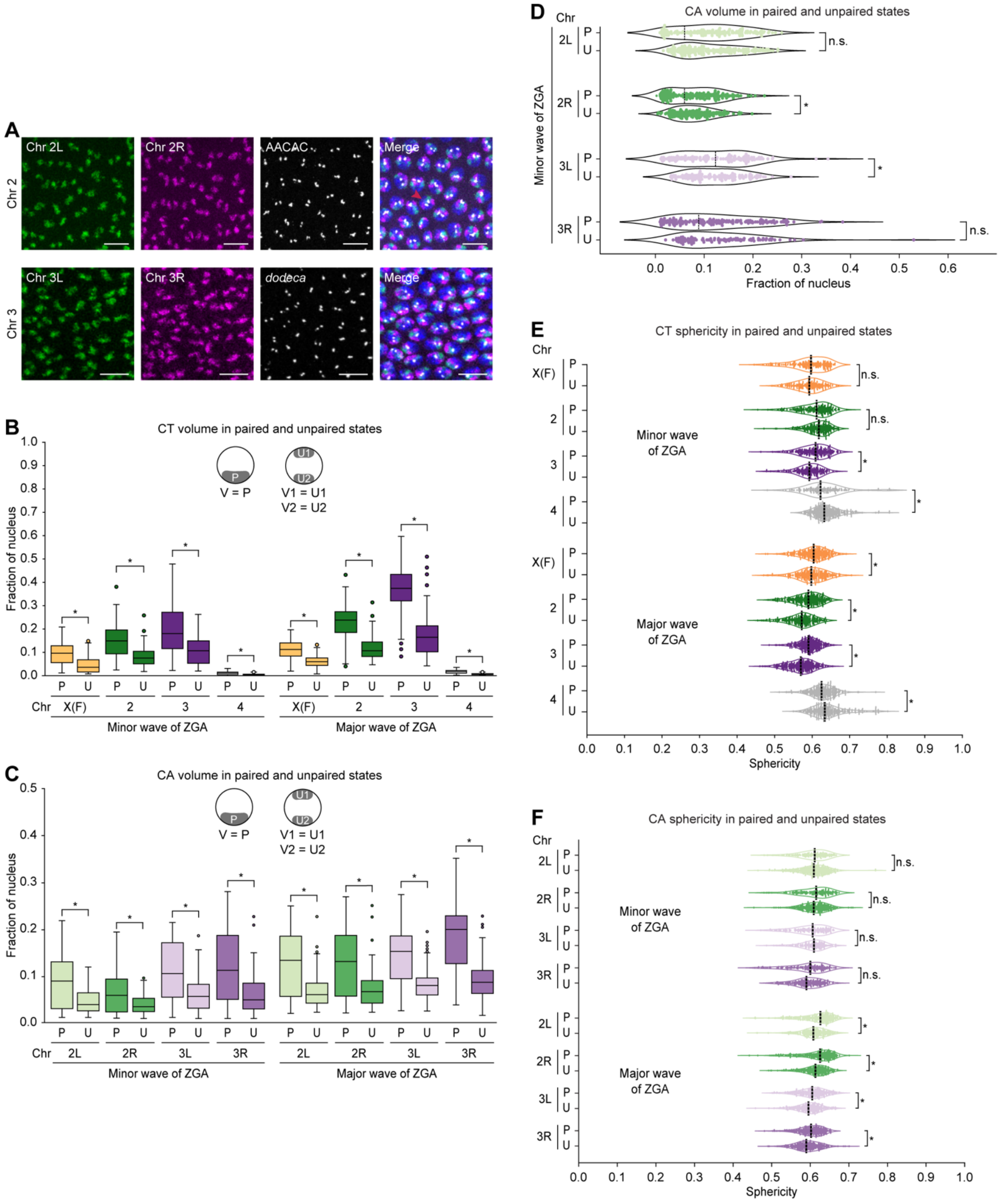

Supplementary Fig. S2. Volume and sphericity changes of CTs and CAs based on pairing.

(**A**) Arms (right, magenta; left, green) of chromosome 2 (top) and 3 (bottom) during major wave of ZGA together with AACAC and *dodeca* probes (gray), respectively. Total DNA by Hoechst stain (blue). Bar = 10 µm. Red arrowhead highlights partial pairing of arms with unpaired centromeric/pericentric regions. (**B**) Normalized CT volume changes between paired homologs to individual unpaired homologs from the minor to major waves of ZGA. X(F), chromosome X in females; P, paired; U, unpaired; U1, unpaired homolog 1; U2, unpaired homolog 2; V, volume; at least three replicates; $n \geq 128$ nuclei; $^*p \leq 1.26\times10^{-9}$, Mann-Whitney two-sided *U* test. (**C**) Normalized volume differences in arms of paired homologs to arms of individual unpaired homologs during ZGA. P, paired; U, unpaired; U1, unpaired homolog 1; U2, unpaired homolog 2; V, volume; at least three replicates; $n \geq 109$ nuclei; $^*p \leq 2.67\times10^{-4}$, Mann-Whitney two-sided *U* test. (**D**) Normalized CA volume differences in arms of paired homologs to the combined volume of two unpaired homologs during ZGA. P, paired; U, unpaired; at least three replicates; $n \geq 103$ nuclei; $^*p \leq 1.12\times10^{-3}$, n.s., not significant, Levene's test. Dashed line indicates local minima for the bimodal distribution observed only in the paired homologs. (**E**) CT sphericity changes between paired and unpaired homologs during ZGA. X(F), chromosome X in females; P, paired; U, unpaired; median, dashed line; at least three replicates; $n \geq 148$ nuclei; $^*p \leq 3.58\times10^{-2}$, n.s., not significant, Mann-Whitney two-sided *U* test. (**F**) CA sphericity of paired and unpaired homologs during ZGA. P, paired; U, unpaired; median, dashed line; at least three replicates; $n \geq 150$ nuclei; $^*p \leq 3.77\times10^{-4}$, n.s., not significant, Mann-Whitney two-sided *U* test; chr, chromosome.

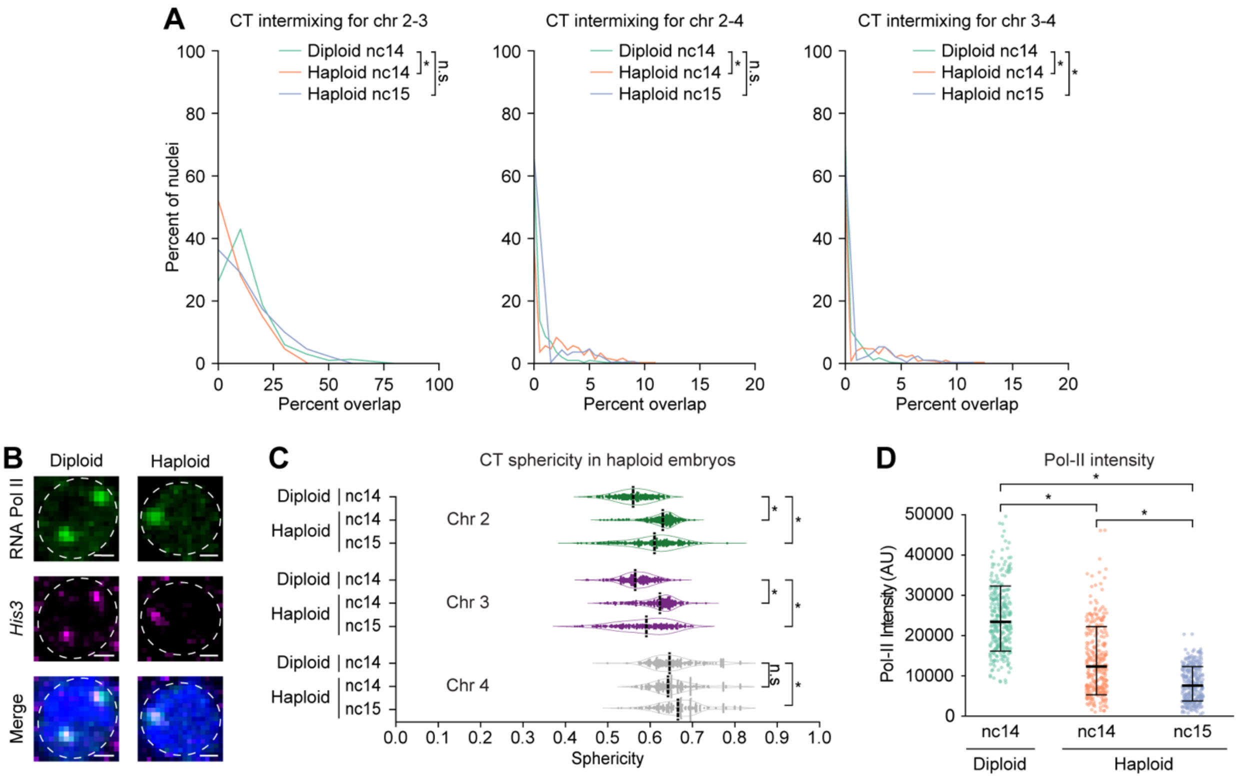

Supplementary Fig. S3. CT intermixing, CT sphericity, and RNA Pol II intensity for diploid and haploid embryos.

(**A**) Histogram with binned data indicating CT intermixing between chromosomes 2, 3, and 4 in diploid and haploid embryos during major wave of ZGA. At least three replicates; n = 300 nuclei; *p ≤ 5.18×10^{-3}, n.s., not significant, Mann-Whitney two-sided *U* test. 2-3 and 2-4 CT overlaps as a percent of chromosome 2 CT volume; chromosome 3-4 overlap as a percent of chromosome 3 CT volume. (**B**) RNA Pol II (green) and *His3* RNA (magenta) in diploid (nc14, left) and haploid embryos (nc15, right) during major wave of ZGA. Bar = 1 µm. (**C**) CT sphericity in diploid and haploid embryos during major wave of ZGA. In diploid embryos, only individual unpaired homolog sphericity was used. Chr, chromosome; median, dashed line; at least three replicates; n ≥ 300 nuclei; *p ≤ 1.87×10^{-7}, n.s., not significant, Mann-Whitney two-

sided *U* test. (**D**) Distribution of RNA Pol II fluorescence intensity (a.u.) within nucleus for diploid and haploid embryos during major wave of ZGA. At least three replicates; $n \geq 300$ nuclei; $*p \leq 3.33\times10^{-18}$, Mann-Whitney two-sided *U* test; nc, nuclear cycle.

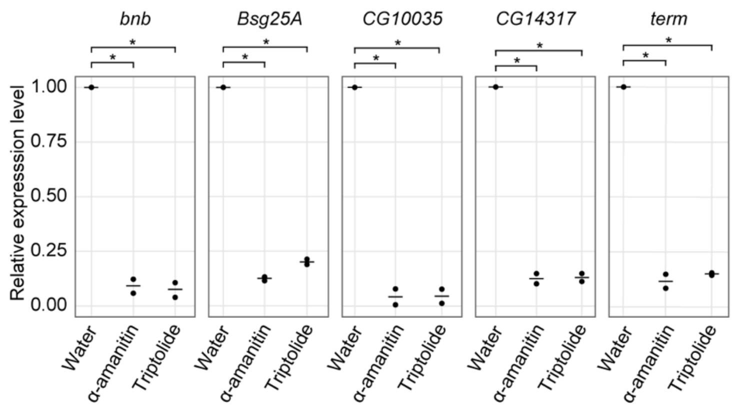

Supplementary Fig. S4. RT-qPCR on microinjected embryos.

RT-qPCR on transcription inhibited embryos showing relative expression level of zygotic transcripts determined by ΔCt value using the maternally-contributed *rp49* transcript as reference. Mean expression level for each condition was normalized to the water-treated samples. Hence, such normalized expression levels in inhibited embryos can be described as portion of transcription relative to the water-treated embryos. Per primer set, RNA was extracted from nc14 embryos across two biological replicates. *$p \leq 1.43\times10^{-3}$, two-tailed Student's t test; mean, solid line.

Supplementary Table S1. Oligopaint summary and CT/CA changes during minor and major waves of ZGA. *See separate excel sheet.*

(**A**) Summary of Oligopaint probes designed. (**B**) Primers for Oligopaint probes. (**C**) Normalized CT volume and (**D**) sphericity during ZGA. (**E**) Normalized CA volume, (**F**) CT intermixing, and (**G**) sphericity during ZGA. X(M), chromosome X in males; X(F), chromosome X in females.

Supplementary Table S2. Pairing of CTs and CAs during ZGA. *See separate excel sheet.*

(**A**) CT pairing and (**B**) CA pairing during minor and major waves of ZGA. (**C**) AACAC and *dodeca* pairing during major wave of ZGA. (**D**) Normalized CT volume and (**E**) CA volume changes between paired homologs to the combined volume of two unpaired homologs. (**F**) Normalized CT volume and (**G**) CA volume differences between paired and individual unpaired homologs. (**H**) CT sphericity and (**I**) CA sphericity between paired and unpaired homologs. P, paired; U, unpaired.

Supplementary Table S3. CT and RNA Pol II dynamics in haploid embryos. *See separate excel sheet.*

(**A**) Nuclear volume, (**B**) normalized CT volume, and (**C**) HLB volume in diploid and haploid embryos during major wave of ZGA. (**B,C**) In diploid embryos, only individual unpaired homolog volumes were used. (**D**) CT intermixing in diploid and haploid embryos. (**E**) CT sphericity differences in diploid and haploid embryos. In diploid embryos, only individual unpaired homolog sphericity was used. (**F**) RNA Pol II fluorescence intensity (a.u.) within nucleus for diploid and haploid embryos.

Supplementary Table S4. CT and nuclear volume measurements in transcription inhibited embryos. *See separate excel sheet.*

(**A**) RT-qPCR primers. (**B**) Nuclear volume, (**C**) normalized CT volume, and (**D**) CT pairing in transcription inhibited embryos during major wave of ZGA.
